# Expression of Concern: MicroRNAs, long non-coding RNAs, and circular RNAs and gynecological cancers: focus on metastasis

**DOI:** 10.3389/fonc.2025.1768138

**Published:** 2026-01-06

**Authors:** Frontiers Editorial Office

With this notice, Frontiers states its awareness of the fact that numerous references in this review have been retracted. Our research integrity team is investigating in full accordance with our procedures. The situation will be updated as soon as the investigation is complete.

UPDATE: This article has now been retracted. Please find the full retraction here: 10.3389/fonc.2026.1967877.

